# Eggs and hatchlings variations in desert locusts: phase related characteristics and starvation tolerance

**DOI:** 10.3389/fphys.2013.00345

**Published:** 2013-12-04

**Authors:** Koutaro O. Maeno, Cyril Piou, Mohamed A. Ould Babah, Satoshi Nakamura

**Affiliations:** ^1^CIRAD, UMR CBGPF-34398 Montpellier, France; ^2^The Mauritanian Desert Locust Centre, Centre National de Lutte AntiacridienneNouakchott, Mauritania; ^3^Japan International Research Center for Agricultural SciencesTsukuba, Japan

**Keywords:** density-dependent phase polyphenism, maternal effect, progeny size and number, locusts, *Schistocerca gregaria*, starvation resistance, risk-spreading strategies

## Abstract

Locusts are grasshopper species that express phase polyphenism: modifying their behavior, morphology, coloration, life history and physiology in response to crowding. Desert locusts, *Schistocerca gregaria*, epigenetically modify progeny quality and quantity in response to crowding. Gregarious (crowded) females produce larger but fewer progeny than do solitarious (isolated) ones. The variability of progeny quality within single egg pod and the reasons why gregarious progeny have a better survival rate than solitarious ones remains unclear. This study investigated 1) the effects of rearing density on the variation in egg size within single egg pods 2) the starvation tolerance of hatchlings from mothers with different phases and 3) the physiological differences in hatchling energy reserve. Isolated females produced smaller but more eggs than did crowded ones. The variation in egg size within egg pods was greater in the latter than in the former. A negative relationship between egg size and number of eggs per egg pod was observed for both groups. Under starvation conditions, gregarious hatchlings survived significantly longer than solitarious ones. Among the solitarious hatchlings, the survival time was longer with increased hatchling body size. However, small individuals survived as long as large ones among the gregarious hatchlings. The percentage of water content per fresh body weight was almost equal between the two phases, before and after starvation. In contrast, the percentage of lipid content per dry body weight was significantly higher in gregarious hatchlings than in solitarious ones before starvation, but became almost equal after starvation. These results demonstrate that female locusts not only trade-off to modify their progeny size and number, but also vary progenies' energy reserves. We hypothesize that gregarious females enhance their fitness by producing progeny differently adapted to high environmental variability and particularly to starvation conditions.

## Introduction

Plasticity and diversification of the characteristics of progeny produced by single individuals appear adaptive to unpredictable and unstable environments as a risk-spreading strategy (Capinera, 1979; Kaplan and Cooper, 1984; Hopper, 1999). Phase polyphenism observed in locusts is a well-known example of reproductive plasticity (Uvarov, 1966; Pener, 1991; Pener and Yerushalmi, 1998; Pener and Simpson, 2009; Verlinden et al., 2009). For example, locusts grown at a low population density (solitarious phase) are characterized by cryptic body coloration and solitary and sedentary habits, whereas those at a high population density (gregarious phase) are characterized by a conspicuous body coloration and gregarious and migratory habits. The variation in these traits is not discrete but continuous, and intermediate forms (transient phase) with intermediate characteristics are observed under certain conditions. In the desert locust, *Schistocerca gregaria* (Forskål), gregarious females occurring in high density populations produce fewer but larger eggs than do the solitarious females that occur in low population densities (Norris, 1952; Uvarov, 1966; Injeyan and Tobe, 1981; Pener, 1991). In the laboratory, the eggs of isolated-reared females typically produce green and small hatchling characteristic of solitarious phase, whereas those laid by crowd-reared ones mainly produce black and large hatchlings characteristic of gregarious phase (Hunter-Jones, 1958). Although the phase-related differences between solitarious and gregarious hatchlings have been frequently reported (Hunter-Jones, 1958; Uvarov, 1966; Bouaïchi et al., 1995; Simpson et al., 1999; Maeno and Tanaka, 2007, 2008a), the variation in progeny size within a single egg pod and the ecological functions of progeny body size are still poorly understood.

In *S. gregaria*, single egg pods laid either by solitarious or gregarious phases have been documented to occasionally contain a mixture of hatchlings with green, intermediate or black body coloration (Faure, 1932; Husain and Ahmad, 1936; Hunter-Jones, 1958; Bouaïchi et al., 1995; McCaffery et al., 1998). This indicates that single egg pods contain different egg sizes, because egg size is closely correlated with hatchling body coloration (Tanaka and Maeno, 2008; Maeno and Tanaka, 2008b). The female desert locusts can flexibly and rapidly modify the quality and quantity of their progeny in response to a change in the rearing density during the adult stage (Bouaïchi et al., 1995; Maeno and Tanaka, 2008a). Timing and duration of crowding experienced by the mothers during a sensitive stage of oocyte development stimulates a shift from the production of small eggs to large ones and vice versa (Maeno and Tanaka, 2010). The primary factor causing progeny gregarization was found to be the tactile stimulus perceived by the antenna (Maeno et al., 2011). Although the mechanisms controlling hatchling characteristics have been addressed from biochemical, physiological and hormonal approaches (Islam et al., 1994; Bouaïchi et al., 1995; McCaffery et al., 1998; Simpson et al., 1999; Hägele et al., 2000; Pener and Simpson, 2009; Maeno and Tanaka, 2012; Van Wielendaele et al., 2012), very scarce information is available regarding the phase-related variation of egg size within a single egg pod in *S. gregaria*. This information becomes essential to understand the mechanism controlling progeny quality and quantity. First, the present study investigated the variation of egg size within egg pods, derived from either isolated- or crowd-reared females, to obtain fundamental information.

Progeny body size influences their developmental and reproductive performance during their own lifetime (Fox and Czesak, 2000). In general, larger progeny exhibit better performance than do smaller sized ones. In *S. gregaria*, the large body size of gregarious hatchlings is likely to be adaptive to adverse conditions, because they show a higher tolerance to desiccation, fasting and poor food conditions than smaller hatchlings of solitarious phase (Albrecht and Blackith, 1960; Maeno and Tanaka, 2011). However, most of the earlier studies investigating the relationship between the phase-related characteristics of the hatchlings and fitness-related performance did not consider the individual variation in hatchling body size. Furthermore, the way in which gregarious hatchlings physiologically accomplish better survival than solitarious ones still remains unclear. Consequently, the adaptive mechanisms of maternal effects on reproduction and progeny fitness in different environments are not well understood (Pener and Simpson, 2009). Therefore, to address these problems, the present study examined the survival of solitarious and gregarious hatchlings individually under starvation conditions. Finally, we examined the physiological changes including the water and lipid contents as the initial energy reserve related to starvation in order to determine the phase-related survival strategies. Investigating the physiological mechanisms involved in producing different qualities of hatchlings from individuals with different maternal histories should help in understanding the ecological role of phase-related characteristics of hatchlings in *S. gregaria*.

## Materials and methods

### Insects and rearing conditions

The *S. gregaria* individuals studied in the present paper were the 6th generation of a new line collected close to Akjoujt (N19°45′, W14°23′) in Mauritania where gregarization of desert locust occur (Babah, 2010). Nymphs and adults were maintained in the Cirad's laboratory, in Montpellier, in groups of approximately 100 individuals in large cages (40 × 40 × 42 cm) or isolated in small cages (12 × 12 × 10 cm) at 31 ± 1°C, with a 12:12 h light:dark photoperiod under 40–60% relative humidity, in a well-ventilated room. They were fed fresh wheat grass leaves as well as wheat bran.

### Effect of rearing density on the egg size, number and variance in egg size within a single egg pod

In *S. gregaria*, rearing density during an adult stage influences the number and size of eggs (Hunter-Jones, 1958; Injeyan and Tobe, 1981; Maeno and Tanaka, 2008a). To confirm this phenomenon for our strain, female locusts reared under crowded conditions as nymphs were placed either under crowded conditions (in a large cage) or isolated in a small boxes, after adult emergence. Each isolated-reared female was paired with a sexually mature male for mating once (<24 h) and allowed to lay egg pods.

Plastic cups (diameter, 5 cm; height, 10 cm) filled with clean moist sand were placed in the cages to collect the egg pods. Egg pods collected during the first two months after adult emergence were incubated at 31 ± 1°C. Two days after egg deposition, eggs were taken one by one, beginning from the lower to the upper portion of the egg pod. These eggs were then placed on a piece of moist tissue paper to avoid desiccation before egg length measurements. Egg length is highly correlated with egg weight (Maeno and Tanaka, 2008b), so the present study measured only egg length to the nearest 0.1 mm using an ocular micrometer installed in a stereo microscope. The number of eggs in each egg pod was counted at that time. Next, all eggs from a single egg pod were placed on moist tissue paper in a plastic container (diameter, 10 cm; height, 10 cm) and returned to the same temperature until hatching. Hatchlings from these egg pods were used for the other experiments described below.

### Relationship between variation in egg size and their position in the egg pod

To determine whether variation in egg size was dependent upon the position in the egg pod, the eggs in each egg pod were divided into three, based on their position of occurrence, i.e., upper, middle and lower regions. All intact eggs within a single egg pod were measured as described above. Curved eggs and eggs broken by handling were not measured but counted.

### Scoring of hatchling body color and body weight

Hatchlings were divided into five hatchling color groups (HCGs 1–5) according to Maeno and Tanaka (2007): HCG 1, green body color without dark spots; HCG 2–4, increasingly darker body color; HCG 5, almost wholly black body color. After body color was scored, the hatchlings were weighed individually to the nearest 0.1 mg with an electronic balance (METTLER AE260). Hatching was observed twice per day (9:00 or 16:00). Due to observation time, the present study used individuals not older than 15 h after hatching. After recording of hatchling body color and body weight, they were used for the starvation experiments described below.

### Maternal effects on starvation tolerance of hatchlings

The starvation experiment was performed using hatchlings obtained from either isolated- or crowd-reared females in the same rearing room as described above. Desert locusts produce typical solitarious and gregarious hatchlings depending on the rearing density during the adult stage, irrespective of the rearing density before the mother reached the adult stage (Hunter-Jones, 1958). If the rearing density of the mother and the progeny are identical (i.e., isolated or crowded), the progeny develop adult morphometrics with the typical solitarious and gregarious morphology, respectively (Maeno and Tanaka, 2009a). For simplification, in this study, hatchlings derived from isolated- and crowd-reared females will be termed solitarious and gregarious hatchlings, respectively. Solitarious and gregarious hatchlings were housed individually in a transparent plastic cylinder (diameter: 3 cm, height: 10 cm) with a piece of moist tissue paper, after being weighed on the day of hatching. Mortality was recorded twice every day. Data from the females and males were pooled.

### Maternal effects on the physiological traits of hatchlings associated with starvation

To determine the influence of starvation on the physiological changes in the hatchlings, fresh body weight within 15 h after hatching, dry body weight, water content as well as lipid content were examined. Two groups were used to measure the total water and lipid contents as physiological characteristics for solitarious and gregarious hatchling. One group included hatchlings before starvation as a control and the other group included those post starvation. Individuals from both groups were weighed on the day of hatching. The hatchlings from the first group were then immediately placed in sealed vials in a freezer (−5°C). In these analyses, we used only hatchlings from the second group that died after 48 h. i.e., only those insects that actually died of starvation. These were placed in the same freezer after weighed at the day of death. Next, the insects were oven-dried at 60°C for 2 days, weighed again and then placed in 1.2 mL chloroform/methanol (2:1) solution for 3 days, during which the solution was changed twice, according to the method of Watanabe and Tanaka (2000). The total water and lipid contents were calculated by the difference between the fresh body weight and dry weight, and between the dry weight and the lean dry weight, respectively. The percentage of water and lipid contents to body size was calculated by water content/fresh body weight, and lipid content/dry body weight, respectively.

### Statistical analysis

The egg lengths and number of eggs per pods were compared between egg pods derived from isolated-reared females and from crowd-reared females using *t*-tests. 1-Way ANOVA and Fisher's PLSD *post-hoc* tests were used to compare the different regions of the egg pods for a given origin. The variances of egg size in a single egg pods were compared between the two groups with *F-test*. Two-Way ANOVAs were used to analyze the effects of starvation and rearing condition on fresh body weight, dry body weight, water content and lipid content. Significant main effects or interactions were examined using Scheffe's *post-hoc* tests. Survival rate of solitarious and gregarious hatchling were compared using a Kaplan-Meier test. Differences in the percentages of water and lipid content relative to the body weight at hatching were analyzed using non-parametric Steel-Dwass test (R Development Core Team, 2012; software package R, version 2.15.0).

## Results

### Effect of rearing density on reproductive traits

**Figure 1:**
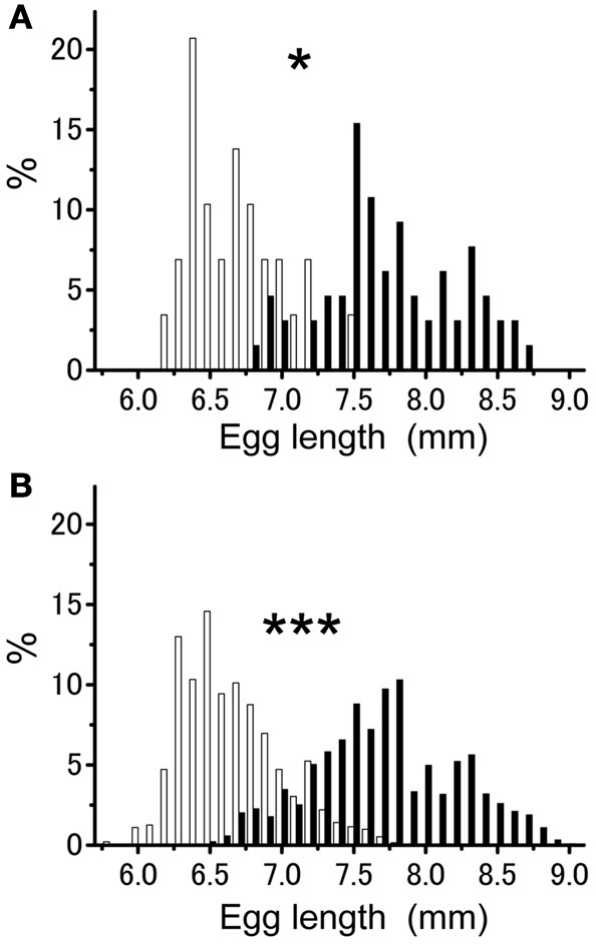
**Percentage of egg sizes within egg pods (A) and for individual eggs (B) produced by the females obtained from either isolated—(white bars; egg pods *n* = 29, individual eggs *n* = 1908) or crowd-reared females (closed bars; egg pods *n* = 65, individual eggs *n* = 3660) in *Schistocerca gregaria*.** Asterisks above the bars indicate significant difference at *P* < 0.001 by *F*-test.

The mean egg length per egg pod for isolated-reared group (mean ± *SE* = 6.69 ± 0.06 mm; *n* = 29) was significantly smaller than that for crowd-reared group (mean ± *SE* = 7.75 ± 0.06 mm; *n* = 65) (Figure 1A; *t*-test; *t* = 11.186, *df* = 1.92; *P* < 0.001). Egg pods laid by isolated-reared females contained eggs ranging in size from 6.2 to 7.5 mm in length (*n* = 29). This range partly overlapped with those obtained from the crowd-reared group (6.8–8.7 mm, *n* = 65). The variance in egg size per egg pod was significantly smaller in the egg pods from isolated-reared females than in those from crowd-reared ones (Figure 1A; *F*-test; *f* = 2.149, *P* < 0.05). A similar tendency was observed when the variances of egg size were compared between the two groups when comparisons of egg size variance were made with values from each group pooled irrespective of egg pod (Figure 1B; *F*-test; *f* = 2.051, *P* < 0.001). However, these significant differences disappeared when considering the coefficient of variation (CV = SD/mean) of the egg size (CV = 0.24 for isolated-reared, CV = 0.25 for crowd-reared, *t*-test, *t* = 0.726, *df* = 92, *P* > 0.05). Both groups had negatively skewed distribution of egg size (mean skewness = −0.303 for isolated-reared and −0.604 for crowd-reared) but were significantly different (*t*-test; *t* = −2.174, *df* = 92, *P* < 0.05).

**Figure 2:**
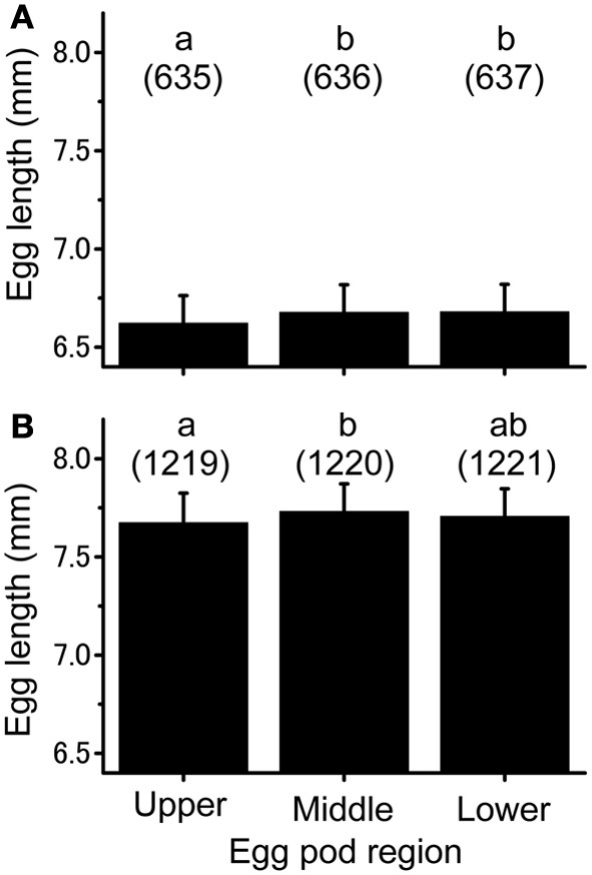
**Individual egg size from different regions of the egg pod laid either by the isolation-reared adults (A) or crowd-reared ones (B) of *Schistocerca gregaria*.** Numbers in parentheses indicate sample sizes. Error bars represent standard-deviations. Different letters above each histogram indicate significant differences at *P* < 0.05 by Fisher's PLSD *post-hoc* test.

Small differences in egg length were observed along the egg pods produced by isolated-reared females (Figure 2A; ANOVA, *f* = 5.47, *df* = 2, *P* < 0.05). Fisher's PLSD test revealed that eggs from the upper region were significantly shorter than those from the middle (*P* < 0.05) and lower (*P* < 0.05) regions. A similar result was obtained for egg pods produced by crowd-reared females, except for the absence of a significant difference between the eggs from the upper and lower parts of the egg pods (ANOVA, *f* = 3.932, *df* = 2, *P* < 0.05; Fisher's PLSD test *P* < 0.05, Figure 2B).

**Figure 3:**
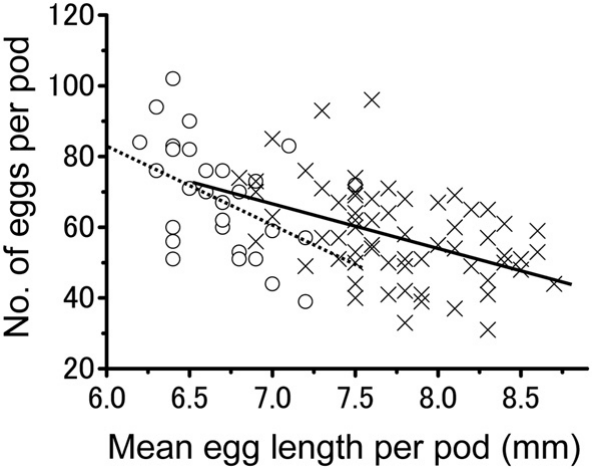
**The relationship between egg size and the number of eggs per pod laid by either the isolation-reared adults (open circles) or crowd-reared *Schistocerca gregaria* (crosses)**. A negative correlation was found for the isolated group (dotted line, *r* = −0.466; *z* = −2.57; *n* = 29; *P* < 0.01) and crowded group (solid line, *r* = −0.444; *z* = −3.75; *n* = 65; *P* < 0.001).

The number of eggs per egg pod was also influenced by rearing density. Egg pods produced by isolated-reared females (mean ± *SE* = 68.8 ± 2.9; *n* = 29) contained significantly more eggs than those produced by their crowd-reared counterparts (mean ± *SE* = 58.0 ± 1.6; *n* = 65) (*t*-test; *t* = −3.460, *df* = 1.92; *P* < 0.001). Figure 3 illustrates the relationship between the egg size and number of eggs per pod produced by either isolated- or crowd-reared females. The overall correlation involving all eggs produced by the two groups was significantly negative (Pearson's correlation; *r* = −0.525; *z* = −5.57; *n* = 94; *P* < 0.001). Similar tendencies were observed for the egg pods produced by isolated-reared females alone (Pearson's correlation; *r* = −0.466; *z* = −2.57; *n* = 29; *P* < 0.01) or those produced by crowd-reared ones (Pearson's correlation; *r* = −0.444; *z* = −3.75; *n* = 65; *P* < 0.001).

### Maternal effects on starvation tolerance of hatchlings

**Figure 4:**
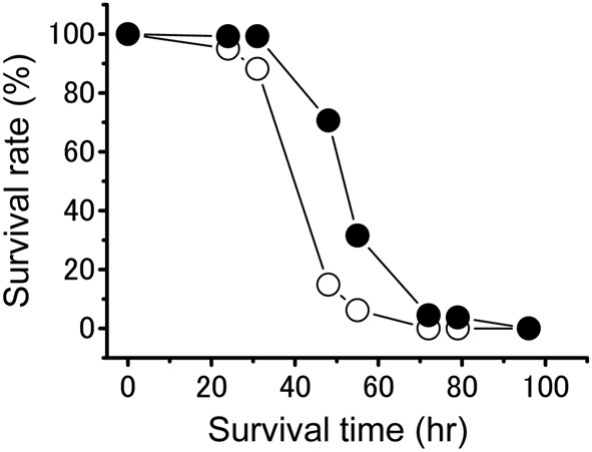
**Survival rate of the solitarious hatchlings (open circle, dotted line: from isolated-reared females) and gregarious ones (closed circle, solid line: from crowd-reared) in *Schistocerca gregaria* under starvation conditions**. Sample size is 161 in the solitarious hatchling group and 133 in the gregarious hatchling group. A Kaplan-Meier test showed a significant difference between the two time series at *P* < 0.001.

The freshly hatched nymphs from isolated-reared females were lighter and greener than the hatchlings from crowd-reared females (Table 1). Phase-related differences were observed in the starvation tolerance of the hatchlings (Figure 4). Gregarious hatchlings produced by crowd-reared mothers survived significantly longer than solitarious ones produced by their isolated-reared counterparts under conditions of starvation (Kaplan-Meier test; *P* < 0.001).

**Table 1:** **Characteristics of body weights (mg ± *SD*) and body coloration of *Schistocerca gregaria* hatchlings categorized into five hatchling color grades (HCGs)**.

**Mother's rearing**		**Body weight at hatching (mg)**			
**Density**	**HCG1**	**HCG2**	**HCG3**	**HCG4**	**HCG5**
Isolation	13.6 ± 1.7	14.8 ± 0.9	17.3 ± 2.0	18.3 ± 1.8	19.1
*N*	132	11	11	6	1
Crowded	15.9	15.0 ± 1.7	16.0 ± 0.7	16.9 ± 0.5	21.1 ± 3.0
*N*	1	6	5	4	117

*Hatchlings in HCG 1 are green as typically observed in solitarious phase and those in HCG 5 are almost completely black as observed in gregarious phase. Those in HCGs 2–4 are intermediate in color and increasingly darker (Maeno and Tanaka, 2007)*.

**Figure 5:**
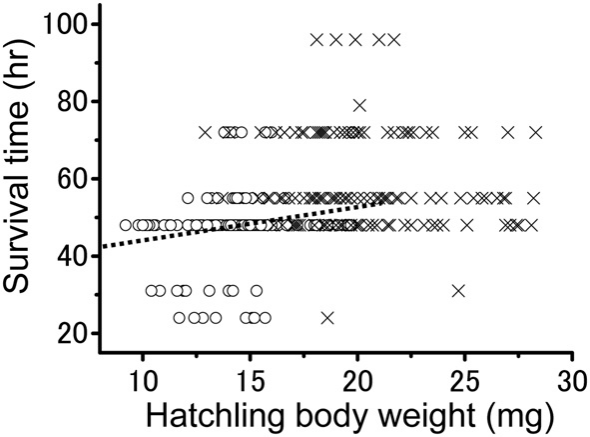
**The relationship between hatchling body weight and survival time in solitarious (open circle: from isolated-reared female) and gregarious hatchlings (crosses: from crowd-reared females) in *Schistocerca gregaria* under starvation conditions**. A positive correlation was found only for the solitarious group (dotted line, *r* = 0.186; *P* < 0.01). *n* = 161 in the solitarious hatchling group and 133 in the gregarious hatchling group.

To investigate the relationship between the survival time and hatchling body weight in more detail, the two values were individually plotted (Figure 5). The overall correlation involving all the hatchlings produced by the two groups was significantly positive (Pearson's correlation; *r* = 0.350; *n* = 294; *P* < 0.001). Survival time was found to significantly increase with body weight at hatching among the solitarious hatchlings (Pearson's correlation; *r* = 0.186; *n* = 161; *P* < 0.01), whereas the value was not significant among those in the gregarious line (Pearson's correlation; *r* = −0.09; *n* = 133; *P* > 0.05). In fact, relatively small gregarious hatchlings survived just as long as the large ones.

### Maternal effects on the physiological traits of hatchlings associated with starvation

**Figure 6:**
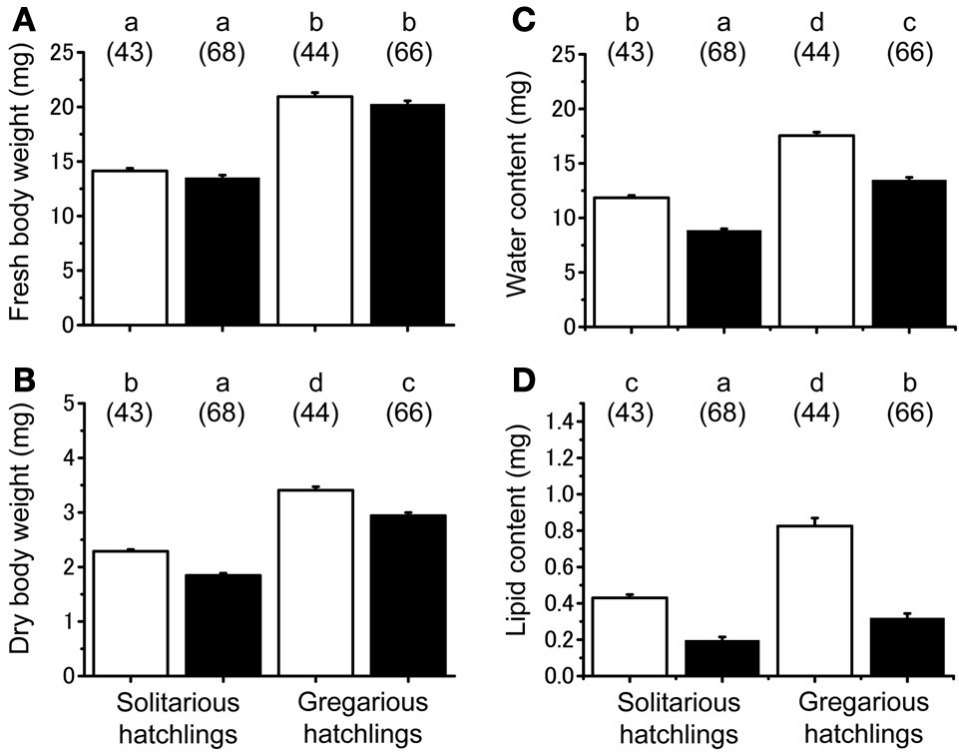
**Physiological changes associated with starvation in hatchlings produced by solitarious hatchings (from isolated-reared mothers) and gregarious ones (from crowd-reared females) in *Schistocerca gregaria*. (A)** Fresh body weight; **(B)** dry body weight; **(C)** water content; **(D)** lipid content. Each variable was measured before (open bar) and after starvation (closed bar). Numbers in parentheses indicate sample sizes. Different letters above each bar indicate significant differences at *P* < 0.05 by Scheffé's *post-hoc* test. Vertical lines indicate *SE*.

**Figure 7:**
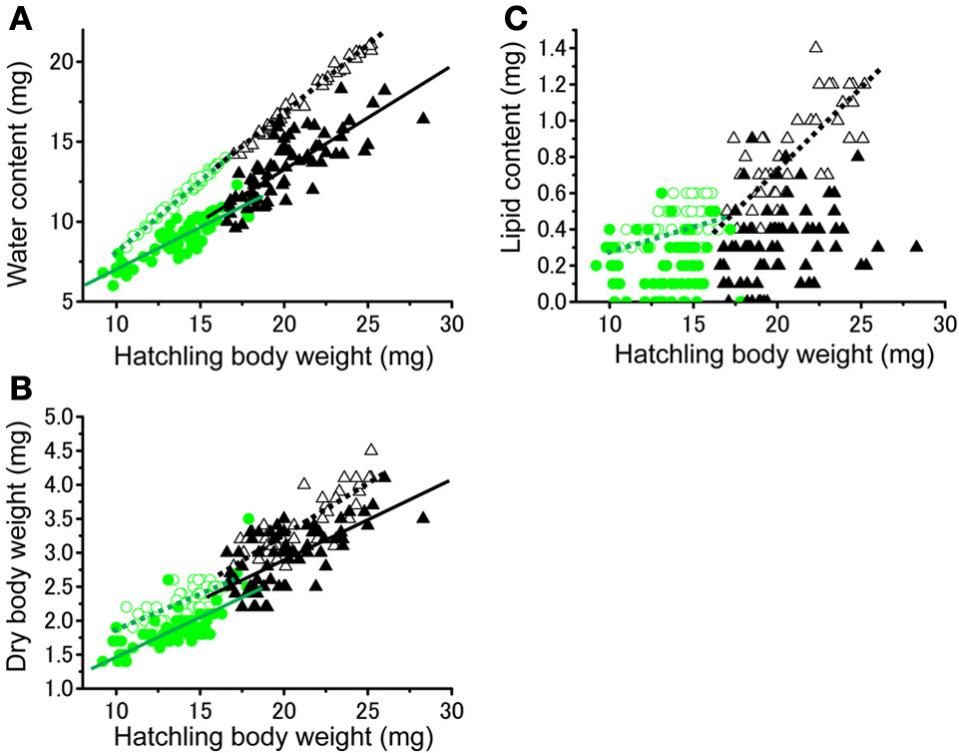
**Relationship between hatchling body weight at hatching and water contents (A), dry body weight (B), and lipid contents (C) of either solitarious (○, •) or gregarious hatchlings (▵, ▴) of *Schistocerca gregaria* pre- (○, ▵) or post- (•, ▴) starvation**. Regression lines are drawn when significant (green for the solitarious, black for gregarious, dotted line for pre-starvation, solid line for post-starvation). See Table 3 for statistical results.

**Figure 8:**
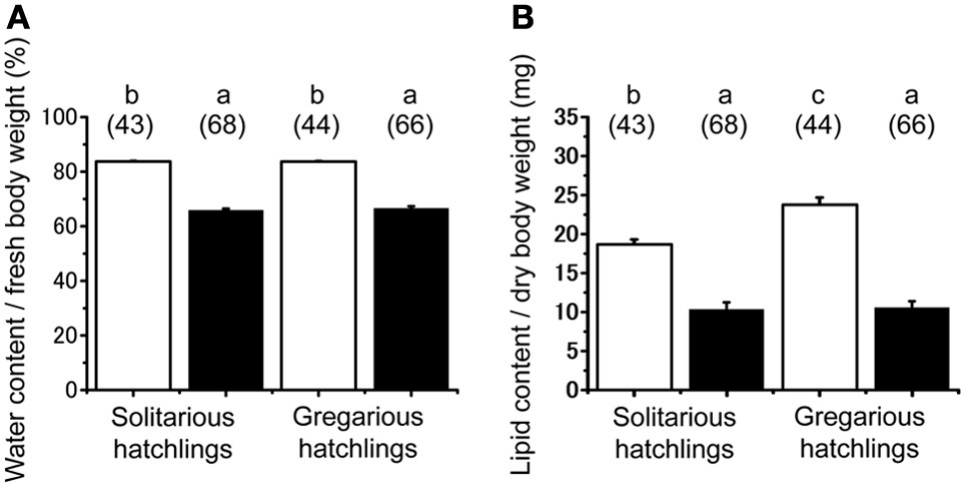
**Starvation-induced physiological changes associated in hatchlings produced by solitarious (isolated-reared mother) and gregarious hatchlings (crowd-reared females) in *Schistocerca gregaria*. (A)** Water content / fresh body weight (mg); **(B)** lipid content/dry body weight (mg). Each variable was measured before (open bar) and after starvation (closed bar). Numbers in parentheses indicate sample sizes. Different letters above each bar indicate significant differences at *P* < 0.05 by Steel-Dwass test. Vertical lines indicate *SE*.

**Figure 9:**
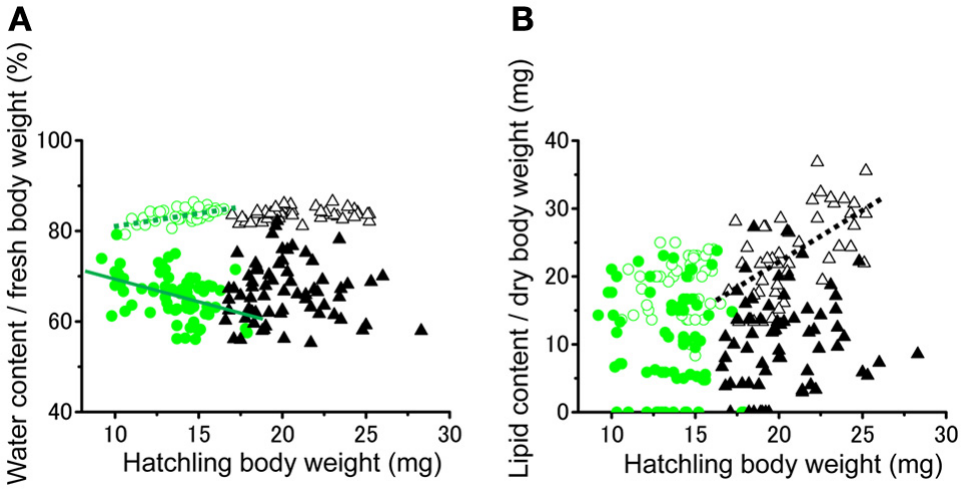
**Relationships between hatchling body weight at hatching and the ratios of percentage of water content / fresh body weight (A) or percentage of lipid content / dry body weight (B) of either solitarious (○, •) or gregarious hatchlings (▵, ▴) of *Schistocerca gregaria* pre- (○, ▵) or post- (•, ▴) starvation**. Regression lines are drawn when significant (green for the solitarious, black for gregarious, dotted line for pre-starvation, solid line for post-starvation). See Table 4 for statistical results.

No significant difference was found in the fresh body weight of the hatchlings, between the hatchlings used for starvation experiments and control for both solitarious and gregarious hatchling groups (*f* = 0.01, *df* = 1, *P* > 0.05, Table 2; Figure 6A). Fresh body weight was significantly greater in gregarious hatchlings than in the solitarious ones (*f* = 507.65, *df* = 1, *P* < 0.001, Table 2; Figure 6A). A similar phase-related difference was observed for dry body weight (*f* = 461.0, *df* = 1, *P* < 0.001, Table 2; Figure 6B), water content (*f* = 471.98, *df* = 1, *P* < 0.001, Table 2; Figure 6C), as well as lipid content (*f* = 87.43, *df* = 1, *P* < 0.001, Table 2; Figure 6D), and the three variables significantly decreased after starvation in both the solitarious and gregarious groups (dry body weight, *f* = 74.20, *df* = 1, *P* < 0.001; water content *f* = 222.25, *df* = 1, *P* < 0.001; lipid content, *f* = 179.57, *df* = 1, *P* < 0.001, Table 2; Figure 6). These three variables were positively correlated to hatchling body weight in both solitarious and gregarious hatchlings, except for the lipid contents of hatchlings after starvation (Table 3; Figure 7). The percentage of water content relative to the fresh body weight at hatching significantly decreased post starvation in both solitarious and gregarious hatchling groups (Figure 8A; Steel-Dwass test; *P* < 0.05); however, the values from the two hatchling groups were almost identical before and after starvation (Steel-Dwass test; *P* > 0.05). Starvation also caused a drop in the percentage of lipid content relative to the dry body weight in both hatchling groups, although the values before starvation were significantly higher in the gregarious hatchlings than in the solitarious ones (Figure 8B). The difference, however, ceased to be significant between the two groups post starvation (Figure 8B; Steel-Dwass test; *P* > 0.05). For solitarious hatchlings, the percentage of water content relative to the fresh body weight at hatching positively correlated with hatchling body weight before starvation, while this tendency became negative after starvation (Figure 9A; Table 4). On the other hand, it was relatively constant for gregarious hatchlings (Figure 9A; Table 4). Hatchling body-weight was correlated to relative lipid content only for gregarious hatchlings pre-starvation (Figure 9B; Table 4).

**Table 2:** **Two way analysis of variance for fresh body weight (mg), water contents (mg), dry body weight (mg) and lipid contents (mg) for *Schistocerca gregaria* hatchlings from either isolated-reared (solitarious) or crowd-reared (gregarious) females**.

**Source of variance**	***df***	**MS**	***f***	***P***
**FRESH BODY WEIGHT**				
Phase	1	2427.61	507.65	<0.001
Starvation	1	23.90	5.00	<0.05
Phase × starvation	1	0.06	0.01	>0.05
Error	217	4.78		
**DRY BODY WEIGHT**				
Phase	1	62.92	461.00	<0.001
Starvation	1	10.13	74.20	<0.001
Phase × starvation	1	0.04	0.30	>0.05
Error	217	0.14		
**WATER CONTENT**				
Phase	1	1404.70	471.98	<0.001
Starvation	1	661.47	222.25	<0.001
Phase × starvation	1	15.27	5.13	<0.05
Error	217	3.00		
**LIPID CONTENT**				
Phase	1	3.53	87.43	<0.001
Starvation	1	7.25	179.57	<0.001
Phase × starvation	1	0.98	24.20	<0.001
Error	217	0.04		

**Table 3:** **Matrices of statistical correlation (Pearson correlation, *r*, *z*-value) between fresh body weight (mg) and water contents (mg), dry body weight (mg) or lipid contents (mg) for *Schistocerca gregaria* hatchlings from either isolated-reared (solitarious) or crowd-reared (gregarious) females**.

**Phase**	**Treatment**	**Water contents (mg)**	**Dry body weight (mg)**	**Lipid contents (mg)**	***n***
Solitarious	Pre-starvation	*r* = 0.994, *z* = 18.10	*r* = 0.693, *z* = 5.40	*r* = 0.375, *z* = 2.49	43
	(control)	^***^	^***^	^*^	
Gregarious	Pre-starvation	*r* = 0.993, *z* = 18.04	*r* = 0.818, *z* = 7.36	*r* = 0.742, *z* = 6.11	44
	(control)	^***^	^***^	^***^	
Solitarious	Post-starvation	*r* = 0.867, *z* = 10.65	*r* = 0.707, *z* = 7.10	*r* = 0.041, *z* = 0.33	68
		^***^	^***^	NS	
Gregarious	Post-starvation	*r* = 0.770, *z* = 8.11	*r* = 0.668, z = 6.40	*r* = 0.235, *z* = 1.90	66
		^***^	^***^	NS	

Hatchlings were exposed to either starvation or not as a control. Pearson correlation: NS, not significant,

* P < *0.05*;

*** *P < *0.001**.

**Table 4:** **Matrices of statistical correlations (Pearson correlation, *r*, *z*-value) between fresh body weight and the ratio of water content/ fresh body weight (%), and between dry body weight and the ratio of lipid content/dry body weight (%) for *Schistocerca gregaria* hatchlings from either isolated-reared (solitarious) or crowd-reared (gregarious) females**.

**Phase**	**Treatment**	**% of water content/fresh body weight (mg)**	**% of lipid content/dry body weight (mg)**	***n***
Solitarious	Pre-starvation	*r* = 0.546, *z* = 3.88	*r* = 0.143, *z* = 0.91	43
	(control)	^***^	NS	
Gregarious	Pre-starvation	*r* = 0.194, *z* = 1.26	*r* = 0.601, *z* = 4.45	44
	(control)	NS	^***^	
Solitarious	Post-starvation	*r* = −0.406, *z* = −3.48	*r* = −0.088, *z* = −0.71	68
		^***^	NS	
Gregarious	Post-starvation	*r* = −0.022, *z* = −0.17	*r* = 119, *z* = 0.95	66
		NS	NS	

Hatchlings were exposed to either starvation or not as a control. Pearson correlation: NS, not significant,

*** *P < *0.001**.

## Discussion

### Density-dependent reproductive characteristics

Crowding experienced by the mother as an adult influences both the size and number of progeny in *S. gregaria*. As reported previously (Norris, 1952; Hunter-Jones, 1958; Uvarov, 1966; Injeyan and Tobe, 1981; Maeno and Tanaka, 2008a), the present study confirmed that our Mauritanian strain of desert locust also exhibit modified egg size and number in response to rearing density. The isolated-reared females produced smaller but more eggs than did the crowd-reared females. We also observed that the variance in egg size in the egg pods was greater when produced by crowd-reared females than when deposited by their isolated-reared counterparts. However, the coefficients of variation were similar for both treatments. These results suggest that variability in egg size between solitarious and gregarious phases is not linked to a phase-specific strategy. We also observed that the skewness of egg size was negative for both phase. These long tails on the left side of the egg-size distributions suggest that there is generally a spread of eggs smaller than the targeted size. This could be interpreted as a common phenomenon that makes a difference between the actual mean egg sizes that the females arrive to get and the optimum that they try to get (the mode of the distribution) depending on their history. The significant difference observed for this skewness is linked to the facts that (1) gregarious females tend to have larger eggs than solitarious ones (making the optimum further away than the actual possible size) but (2) small failed eggs exist for both (spreading the distribution more for the gregarious group). These observations argue for physiological processes that selectively allow gregarious females to produce bigger eggs but do not argue for an additional risk-spreading strategy (i.e., bet-hedging; Hopper, 1999) that gregarious females would use by varying their eggs' sizes.

Mixtures of characteristics of solitarious and gregarious hatchlings appearing from single egg pods were previously documented (Faure, 1932; Husain and Ahmad, 1936; Hunter-Jones, 1958; Bouaïchi et al., 1995; McCaffery et al., 1998; Tanaka and Maeno, 2006). Different hatchlings may come from different egg sizes within the egg pods, because the degree of darkness of the body is correlated with egg size (Tanaka and Maeno, 2008). The present study supports this idea and provides another physiological factor that may contribute to the effect: the distribution of sizes within the egg pod. However, this assumes that all ovarioles do not produce eggs of the same size simultaneously.

The present study observed that the variation in egg size was larger in the egg pods produced by crowd-reared females than those by isolated ones. Phase-related reproductive traits could explain the different degrees of variation in egg size between the two groups. In *S. gregaria*, the longer the duration of the interval between ovipositions the bigger the mean egg size per egg pod (Maeno and Tanaka, 2009b). Crowded conditions tend to increase the interval between ovipositions and therefore also increase egg size (Maeno and Tanaka, 2009b). Although the present study did not record the ovipositional interval individually under crowded conditions, there is a possibility that such a long ovipositional interval could be a factor that also influences the large variation in egg size within a single egg pod. Different oocyte sizes were observed among the ovarioles during oocyte development, for both the solitarious and gregarious females (Maeno, unpublished observations). Differences in egg development among the ovarioles also appear to cause a variation in egg size. Further study is necessary to better understand the physiological mechanisms controlling progeny quality and quantity.

In *S. gregaria*, a trade-off between progeny size and number was observed for the solitarious locusts, although not for the gregarious ones (Maeno and Tanaka, 2008a). In contrast, the present study found a trade-off even for egg pods produced by gregarious (crowd-reared) females. The discrepancy between the two studies can be explained by variations in the reproductive cycle. The first egg pods produced by the gregarious locusts contained substantial numbers of small eggs which in turn produced green hatchlings typical of the solitarious (isolated-reared) phase, whereas those deposited after the first egg pod predominantly produced black hatchlings, typical of the gregarious phase (Maeno and Tanaka, 2008b). Although the present study did not record the reproductive cycle, all the egg pods collected were used for the analysis. The earlier study did not use the first egg pods produced by the crowd-reared females for analysis (Maeno and Tanaka, 2008a), which may explain why they did not observe the trade-off. The process by which egg size and egg number per clutch are controlled in the ovary remains unclear. Desert locusts allometrically modify their egg size and number in response to a change in rearing density (Maeno and Tanaka, 2008a). The number of eggs decreased with increased egg size when isolated-reared females were exposed to crowding (Maeno and Tanaka, 2008a). Juvenile hormone was long believed to cause reproductive solitarization (i.e., producing smaller and more eggs) (Pener, 1991), but more recent work suggests it may not be involved (Maeno and Tanaka, 2009b; Verlinden et al., 2009).

### Phase-related progeny fitness and physiological traits of hatchlings associated with starvation

Progeny body size influences fitness-related performance in insects (Fox and Czesak, 2000). Large gregarious hatchlings showed better survival rate and developmental performance than small solitarious ones (Maeno and Tanaka, 2008b, 2011). The present study confirmed the observation by Albrecht and Blackith (1960) that under starvation conditions gregarious hatchlings with large body size survived longer than solitarious ones with small body size. Additionally, the present study further demonstrated that the gregarious hatchlings showed specific body size-dependent survival patterns. Among the solitarious hatchlings, the survival time increased proportionally to the increase in body weight as generally observed, whereas the relatively small gregarious hatchlings survived as long as the large ones. In fact, even though the range of body sizes was greater in gregarious hatchlings, the survival time was almost constant, suggesting that gregarization in hatchlings might be related to fitness-related survivorship.

The initial energy supply of hatchlings was determined by the mothers. Thus far, lipids have been considered the main resource of energy not only for embryonic development but also for hatchlings (Arrese and Soulages, 2010). Our lipid content measures and the difference among solitarious and gregarious hatchlings were in agreement with Blackith and Howden (1961) except for the range of values, which probably is due to different techniques.

The present study demonstrated that the energy reserve and utilization patterns differed between solitarious and gregarious hatchlings. The initial percentage of lipid relative to body weight was greater in the gregarious hatchlings than in the solitarious ones. Among gregarious hatchlings, this proportion increased as body size increased; however, after starvation it became equal to that of solitarious hatchlings. The initial high percentage of lipid content relative to the body size in the gregarious hatchlings could play an important physiological role to enhance survival under certain conditions. Hatchlings began to walk actively in the experimental cylinder when they were hungry (data not shown). Gregarious hatchlings are well known to be more active than solitarious ones (Ellis and Pearce, 1962; Uvarov, 1966; Bouaïchi et al., 1995; Simpson et al., 1999; Hoste et al., 2006; Harano et al., 2012), and it has been presumed that hatchlings' activity and metabolism rate are positively correlated to body size. Although the present study did not measure the total activity of hatchlings, there is a possibility that the survival time of the large gregarious hatchlings was limited by their faster metabolism of energy reserves compared to that by small gregarious hatchlings. However, Blackith and Howden (1961) suggested that non-fatty substances also may act as energy sources.

In *S. gregaria*, water is absorbed by the egg itself during embryonic development (Shulov and Pener, 1963). Blackith (1961) reported that the longer survival of the large unfed hatchlings is attributable to their greater initial water reserves. The present study confirmed that water was consumed during starvation, although the percentage of water weight relative to fresh body weight was almost identical between the solitarious and gregarious hatchlings. The utilization pattern of water was body-size dependent for solitarious hatchlings, but not for gregarious ones: larger solitarious hatchlings had higher percentage of water relative to body weight, whereas no significant correlation was found among gregarious hatchlings. These different physiological changes might not be explained by only body size, but phase-specific characteristics.

## Conclusion

In conclusion, the maternal response to crowding, by increasing progeny size and the degree of variation in progeny size via trade-offs between progeny quality and quantity, appeared to be an advantageous method to cope with the adverse environments as observed during outbreak. In *S. gregaria*, gregarious adult females aggregate together and lay eggs in groups (Stower et al., 1958). Hatchlings from these eggs would sometimes suffer from unpredictable severe competition for food resources because the vegetation would have already been damaged by their parents. Also, conspecific individuals would synchronously hatch within the limited area, and might have little choice of food plants. Under such conditions, it would be beneficial for the female to produce large gregarized hatchlings that carry relatively large amounts of lipid as energy reserve conferring them high survivorship irrespective of their size. Consistent with this idea, a high percentage of gregarious progeny demonstrate greater tolerance for starvation and greater locomotion, thus increasing the likelihood of survival until they can reach an alternate food source. On the other hand, the solitarious hatchlings are likely to find themselves in areas where vegetation is available (Uvarov, 1977). Increasing the reproductive resource allocation to the number of progeny instead of to size as observed for the isolated-reared females appears to be a favorable strategy when the survival between the small and large hatchlings is almost similar. Such reproductive and survival strategies adopted by the desert locusts could play an important role in contributing to the population growth. Kaplan and Cooper (1984) predicted that with variable environments, traits allowing variable offspring size are selected. Desert locusts are widely distributed and migrate over long distances, dealing with various types of environments (Uvarov, 1977), which could have favored the evolution of variability in the phase-related strategies.

### Conflict of interest statement

The authors declare that the research was conducted in the absence of any commercial or financial relationships that could be construed as a potential conflict of interest.
